# Supplementary material for: σE of Streptomyces coelicolor can function both as a direct activator or repressor of transcription
Source: Commun Biol. 2024 Jan 6;7:46. doi: 10.1038/s42003-023-05716-y (PMC10771440; doi:10.1038/s42003-023-05716-y)
Supplement: Supplementary file 2 — Supplementary Information [file 42003_2023_5716_MOESM2_ESM.pdf]

SUPPLEMENTARY INFORMATION

σ^E of *Streptomyces coelicolor* can function both as an activator and repressor of transcription

Jiří Pospíšil, Marek Schwarz, Alice Ziková, Dragana Vítovská, Miluše Hradilová, Michal Kolář, Alena Křenková, Martin Hubálek, Libor Krásný, Jiří Vohradský

Supplementary Figures 1-4
Supplementary Table 1
Supplementary Tables 2-6 captions
Supplementary References

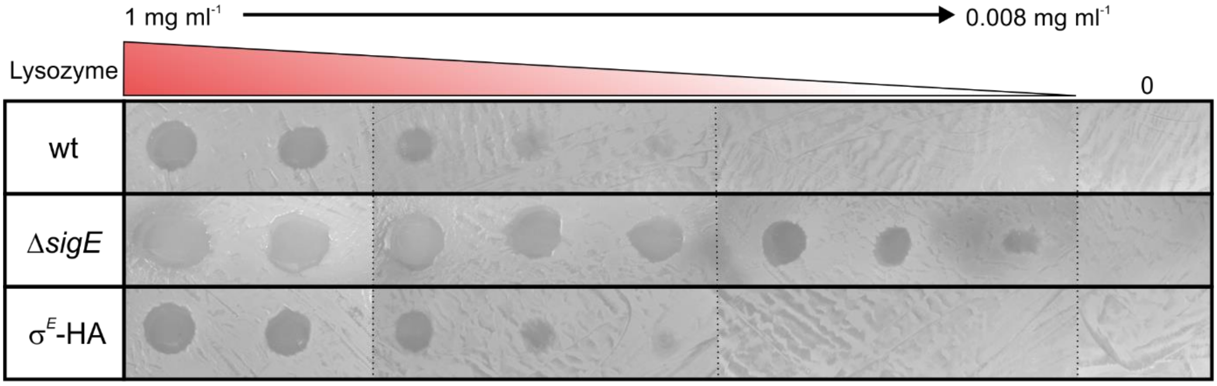

Supplementary Figure 1. Lysozyme sensitivity assay

S. coelicolor strains LK3801 (wt), LK3804 (Δ*sigE*) and LK3802 (σ^E-HA) were tested for lysozyme sensitivity. Two-fold dilution series of lysozyme (the gradient and concentrations are indicated) was spotted on freshly platted bacterial lawn (from spore suspension). Pictures were taken after two-day incubation at 30 °C when zones of clearing were visible. The experiment was performed 2-times with identical results. All zones of clearing in one line come from one plate; the dotted lines indicate sites of their electronical assembly (originally on the plate, the zones were not in one straight line).

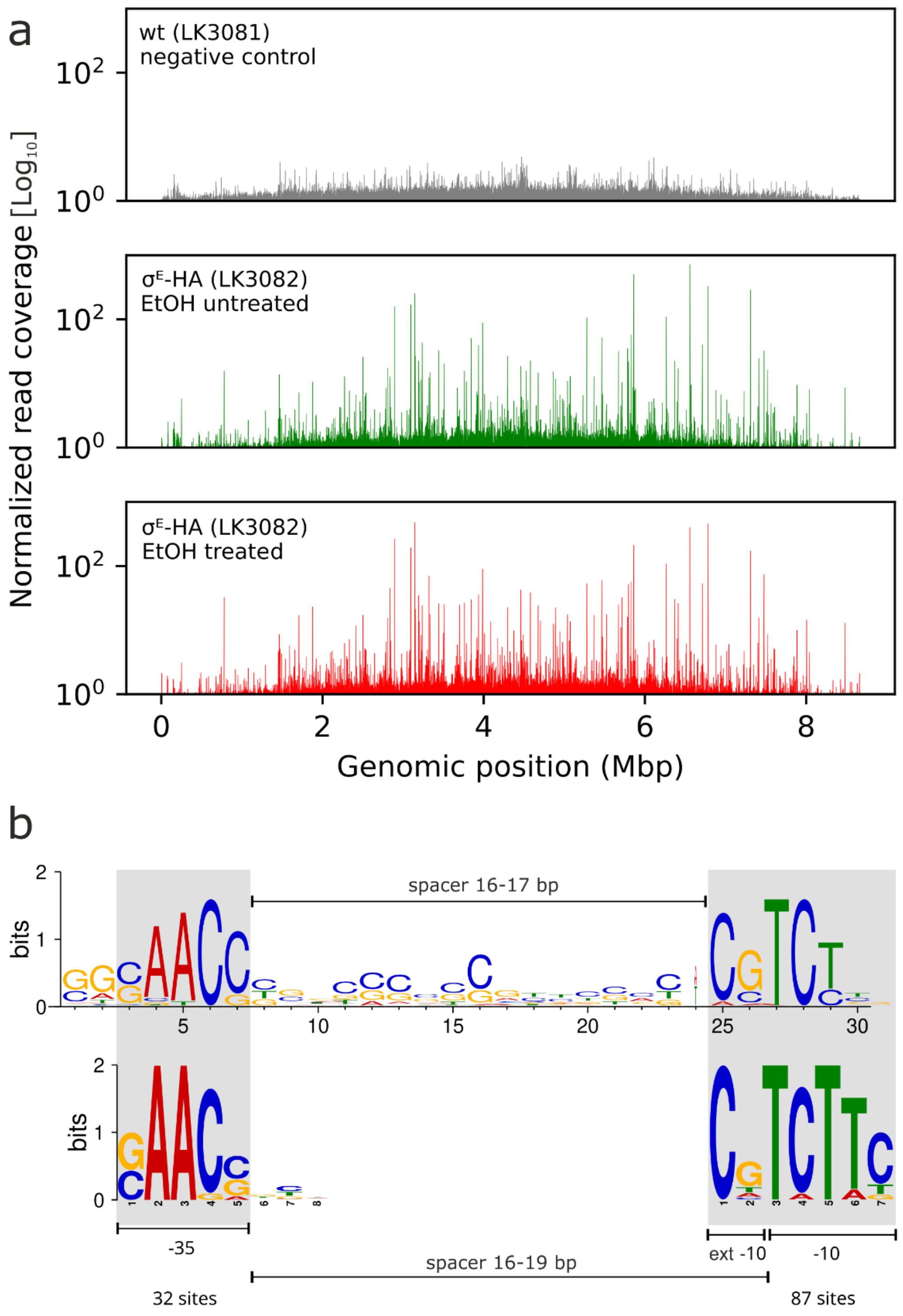

Supplementary Figure 2. Overview of normalized genome coverage for ChIP-seq and comparison of sequence logo

a) The overview of read coverage for each condition across whole genome. Reads from each condition were mapped with Bowtie2 and coverage was computed with deepTools2 bamCoverage with RPGC normalization with effective genome size computed by Kent's tools faCount. The y-axis represents the number of reads per 1 nucleotide/scaling factor for 1x average coverage.

b) Comparison of σ^E binding motifs described by Tran *et. al.* [1](upper motif)] with the two conserved half-sites discovered in this work.

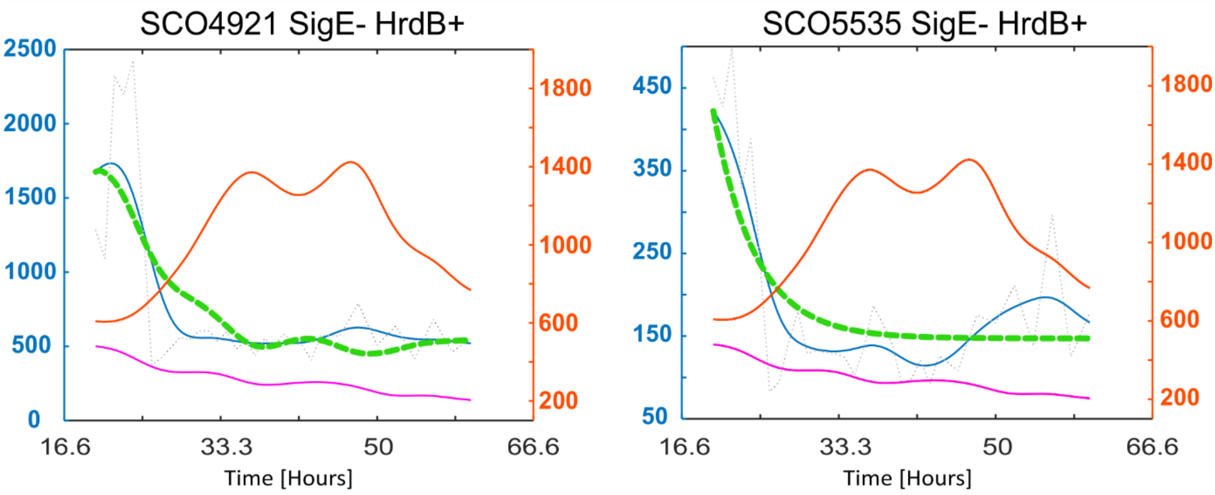

Supplementary Figure 3. Kinetic modeling of SCO4921 and SCO5535.

The gray dotted line represents original transcriptomic data. A curve fitted to the data is shown in blue, kinetic modeling results in green σ^E in orange, HrdB in magenta. Blue and orange vertical axes represent relative expression of regulated gene and regulators, respectively. Signs (+) or (–) in descriptions above the charts indicate activation or repression.

Goodness-of-fit of computational modeling (numbers in following brackets) for SCO4921 was lower for SigE- HrdB+ (24) than for HrdB+ alone (140) showing better modeling results (better fit of green line to regulated gene – blue line). Similar results were obtained for SCO5535 regulated by SigE- HrdB+ (29) vs. SigE- alone (32). For comparison, see computational modeling of SCO4921 and SCO5535 only by HrdB as an activator in Figure 5.

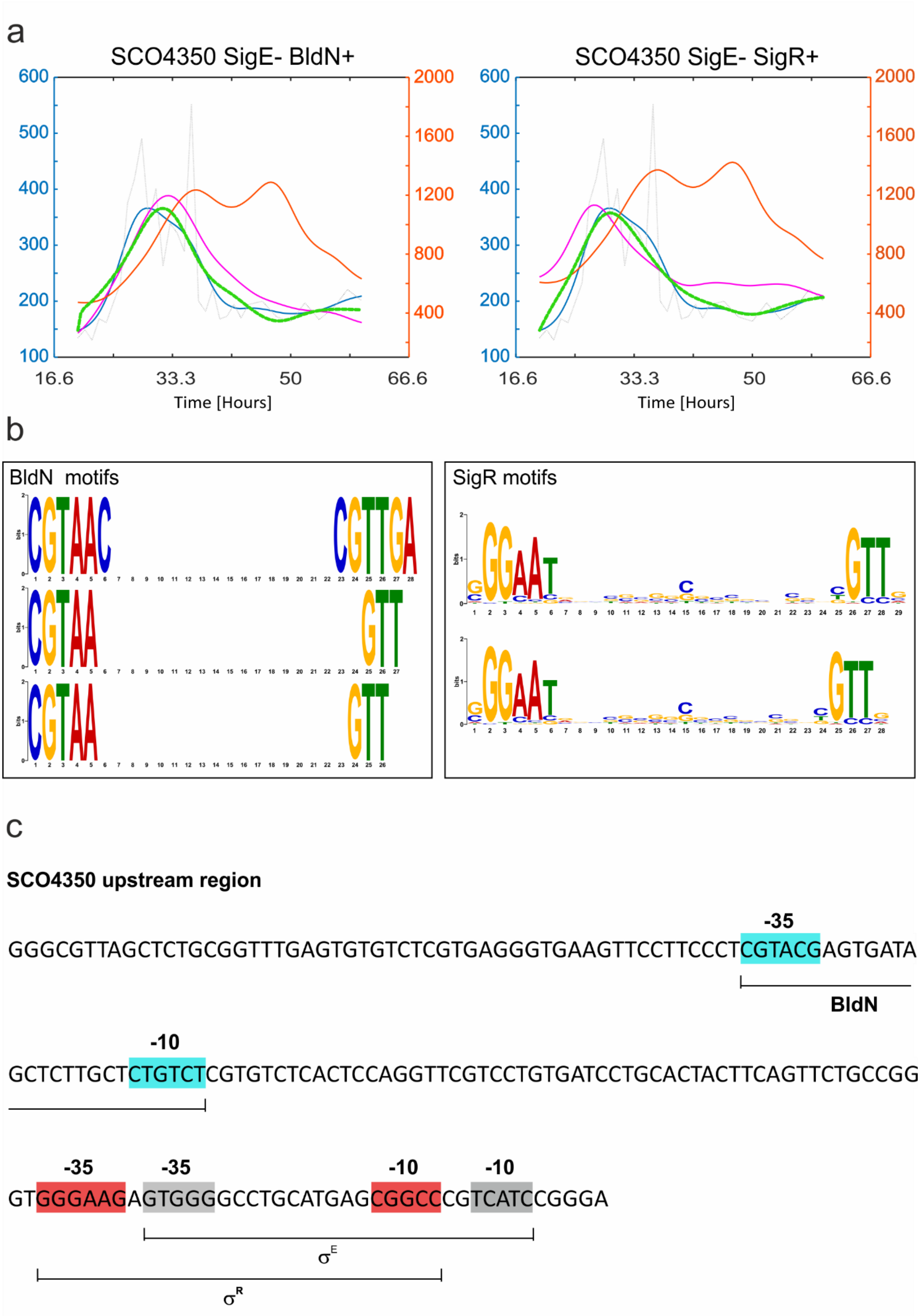
a
SCO4350 SigE- BldN+
SCO4350 SigE- SigR+
600
500
400
300
200
100
2000
1600
1200
800
400
16.6
33.3
50
66.6
Time [Hours]
b
BldN motifs
SigR motifs
c
SCO4350 upstream region
-35
GGGCGTTAGCTCTGCGGTTTGAGTGTGTCTCGTGAGGGTGAAGTTCCTTCCCTCGTACGAGTGATA
BldN
-10
GCTCTTGCTCTGTCTCGTGTCTCACTCCAGGTTCGTCCTGTGATCCTGCACTACTTCAGTTCTGCCGG
-35
-35
-10
-10
GTGGGAAGAGTGGGGCCTGCATGAGCGGCCCGTCATCCGGGA
σE
σR

Supplementary Figure 4. Regulation of SCO4350.

a) The gray dotted line represents original transcriptomic data. A curve fitted to the data is shown in blue, kinetic modeling results in green σ^E in orange, BldN (left graph) and (σ^R-right graph) in magenta. Blue and orange vertical axes represent relative expression of the regulated gene and regulators, respectively. Signs (+) or (–) in descriptions above the charts indicate activation or repression. Goodness-of-fit of computational modeling (numbers in following brackets) for SigE- BldN+ (24) and SigE- SigR+ (13.7) were lower than for SigE+, (56) indicating better modeling results (better fit of green line to the regulated gene – blue line). For comparison, see computational modeling of SCO4350 only by SigE- as a repressor in Figure 4.

b) Binding motifs of BldN and σ^R that were used for analysis of the SCO4350 upstream region. The BldN motifs are based on reported consensus sequences[2,3]. The σ^R motifs (differing in spacer length) are based on 58 σ^R-dependent binding sites defined in a previously published ChIP-chip experiment[4].

c) Sequence of the SCO4350 promoter and its upstream region. Predicted consensus sequences are highlighted in cyan, red, and grey for BldN, σ^R, and σ^E respectively. The *p-valu*e of the sequence prediction is < 0.001. This DNA sequence was used in the promoter activity assay (see Fig. 7B).

Strain	Genotype	Source
S. coelicolor		
LK3801	SCP1- SCP2- A3(2) M145	[5]
LK3802	A3(2) M145 *sigE*-HA::*apr*	This work
LK3803	SCP1- SCP2- A3(2) M600	[6]
LK3804/J2130	Δ*sigE,* M600	[6]
LK3626	A3(2) M145/pBPSA1-$P_{SCO7657}$-*bpsA::apr*	This work
LK3630	A3(2) M145/pBPSA1-$P_{SCO7657-SigEmut}$-*bpsA::apr*	This work
LK3633	A3(2) M145/pBPSA1-$P_{SCO4350}$-*bpsA::apr*	This work
LK3635	A3(2) M145/pBPSA1-$P_{SCO4350- SigEmut}$-*bpsA::apr*	This work
LK3716	A3(2) M145/pBPSA-*bpsA::apr*	This work
E. coli		
ET12567	*dam-13*::Tn*9, dcm-6, hsdM*	[7]
BW25113	*lacI*^{q} *rrnB*$_{T14}$ Δ*lacZ*$_{WJ16}$ *hsdR514* Δ*araBA-D*$_{AH33}$ Δ*rhaBAD*$_{LD78}$	[8]
**Plasmids/cosmids**	**Description**	**Source**
pBPSA1	*aac(3)IV*,*apr*, *oriT, tfd, bpsA, λt0*	[9]
pIJ773	*aac(3)IV*,*apr*, *oriT*	[10]
pIJ790	λ-RED (*gam, bet, exo*), *cat, araC, rep101*^{ts}	[10]
pUZ8002	*tra, neo,* RP4	[6]
StE94	*carb, kan*	Cosmid library, Earlham Institute, Norwich, UK
StE94/*sigE*-HA	*carb, kan, sigE*-HA::*apr, oriT*	This work

Supplementary Table 1. **Strains and plasmids used in this study.**

Supplementary Tables 2-6 captions

Supplementary Tables 2-6 can be found in Supplementary Data 6.

Supplementary Table 2. List of primers used in this study.

Supplementary Table 3. Genes identified by ChIP-seq experiment.

Genes that were found exclusively in EtOH stress experiments and not in non-stressed conditions are highlighted with green color. The only significantly overrepresented functional group was the Periplasmic/exported/lipoproteins group (hypergeometric test $p<0.05$). The overall consensus sequence (xAACxxxxxxxxxxxxxxxxxxCxTCTxx) denotes nucleotides with occurrence > 70% at respective position in identified motif sites adjusted to same width by inserting "-" before the terminal octamer when needed; "x" denotes ambiguous position. The Identity to consensus was then computed as a fraction of the number of nucleotides in the identified motif matching the consensus nucleotides.

Supplementary Table 4. Group A, σ^E -dependent genes identified in this study.

Genes that were found exclusively in EtOH stress experiments and not in non-stressed conditions are highlighted with green color. Genes found also by Tran *et al.*[1] are highlighted with orange. Genes with blank background were exclusively identified in this study. All genes were preceded by a σ^E binding motif. SCO - gene identifier, σ^E model – σ^E kinetic model found, HrdB motif – HrdB binding motif found, HrdB model – HrdB kinetic model found, HrdB peak – peak found in ChIP-seq of HrdB experiment[11]. The group consensus sequence (xAACxxxxxxxxxxxxxxxxxxCxTCTxx) denotes nucleotides with occurrence > 70% at respective position in identified motif sites adjusted to same width by inserting "-" before the terminal octamer when needed; "x" denotes ambiguous position. The Identity to consensus was then computed as a fraction of the number of nucleotides in the identified motif matching the consensus nucleotides.

Supplementary Table 5. Group B, HrdB-dependent genes identified in this study.

Genes also found by Tran *et al.*[1]are highlighted with orange. Genes with blank background were exclusively identified in this study. All genes had a σ^E binding motif. SCO - gene identifier, σ^E model – σ^E kinetic model found, HrdB motif – HrdB binding motif found, HrdB model – HrdB kinetic model found, HrdB peak – peak found in ChIP-seq of HrdB experiment[11]. The group consensus sequence (xAxCxxxxxxxxxxxxxxxxxxCxTCxxx) denotes nucleotides with occurrence > 70% at respective position in identified motif sites adjusted to same width by inserting "-" before the terminal octamer when needed; "x" denotes ambiguous position. The Identity to consensus was then computed as a fraction of the number of nucleotides in the identified motif matching the consensus nucleotides.

Supplementary Table 6. Group C- genes with flat profiles identified in this study.

Genes that were found exclusively in EtOH stress experiments and not in non-stressed conditions are highlighted with green color. Genes also found by Tran *et al.*[1] are highlighted with orange. Genes with blank background were exclusively identified in this study. All genes had a σ^E binding motif. SCO - gene identifier, HrdB motif – HrdB binding motif found, HrdB model – HrdB kinetic model found, HrdB peak – peak found in ChIP-seq of HrdB experiment[11]. The group consensus sequence (xAACxxxxxxxxxxxxxxxxxxCGTCTxx) denotes nucleotides with occurrence > 70% at respective position in identified motif sites adjusted to same width by inserting "-" before the terminal octamer when needed; "x" denotes ambiguous position. The Identity to consensus was then computed as a fraction of the number of nucleotides in the identified motif matching the consensus nucleotides.

Supplementary References

1. Tran, N. T. *et al.* Defining the regulon of genes controlled by σE , a key regulator of the cell envelope stress response in Streptomyces coelicolor. *Mol Microbiol* **112**, 461–481 (2019).
2. Bibb, M. J., Molle, V. & Buttner, M. J. σ(BldN), an Extracytoplasmic Function RNA Polymerase Sigma Factor Required for Aerial Mycelium Formation in Streptomyces coelicolor A3(2). *J Bacteriol* **182**, (2000).
3. Touzain, F. *et al.* SIGffRid: A Tool to Search for Sigma Factor Binding Sites in Bacterial Genomes Using Comparative Approach and Biologically Driven Statistics. *BMC Bioinformatics* **9**, 73 (2008).
4. Kim, M. S. *et al.* Conservation of Thiol-oxidative Stress Responses Regulated by SigR Orthologues in Actinomycetes. *Mol Microbiol* **85**, (2012).
5. Bentley, S. D. *et al.* Complete genome sequence of the model actinomycete Streptomyces coelicolor A3(2). *Nature* **417**, 141–147 (2002).
6. Paget, M. S. B., Chamberlin, L., Atrih, A., Foster, S. J. & Buttner, M. J. Evidence that the Extracytoplasmic Function Sigma Factor E Is Required for Normal Cell Wall Structure in Streptomyces coelicolor A3(2). *J Bacteriol* **181**, 204–211 (1999).
7. Flett, F., Mersinias, V. & Smith, C. P. High efficiency intergeneric conjugal transfer of plasmid DNA from Escherichia coli to methyl DNA-restricting streptomycetes. *FEMS Microbiol Lett* **155**, 223–229 (2006).
8. Datsenko, K. A. & Wanner, B. L. One-step inactivation of chromosomal genes in Escherichia coli K-12 using PCR products. *Proceedings of the National Academy of Sciences* **97**, 6640–6645 (2000).
9. Knirschova, R. *et al.* Utilization of a reporter system based on the blue pigment indigoidine biosynthetic gene bpsA for detection of promoter activity and deletion of genes in Streptomyces. *J Microbiol Methods* **113**, 1–3 (2015).
10. Gust, B., Challis, G. L., Fowler, K., Kieser, T. & Chater, K. F. PCR-targeted Streptomyces gene replacement identifies a protein domain needed for biosynthesis of the sesquiterpene soil odor geosmin. *Proceedings of the National Academy of Sciences* **100**, 1541–1546 (2003).
11. Šmídová, K. *et al.* DNA Mapping and Kinetic Modeling of the HrdB Regulon in Streptomyces coelicolor. *Nucleic Acids Res* **47**, 621–633 (2019).
