# Supplementary material for: σE of Streptomyces coelicolor can function both as a direct activator or repressor of transcription
Source: Commun Biol. 2024 Jan 6;7:46. doi: 10.1038/s42003-023-05716-y (PMC10771440; doi:10.1038/s42003-023-05716-y)
Supplement: Supplementary file 3 — Description of Supplementary Materials [file 42003_2023_5716_MOESM3_ESM.docx]

**Description of Additional Supplementary Files**

**File name:** Supplementary Data 1

**Description:** ChIP-seq peaks and binding motifs.

**File name:** Supplementary Data 2

**Description:** Expression series.

**File name:** Supplementary Data 3

**Description:** Model parameters.

**File name:** Supplementary Data 4

**Description:** SigE regulons comparison.

**File name:** Supplementary Data 5

**Description:** Source Data.

**File name**: Supplementary Data 6

**Description:** Supplementary Tables 2-6.
