# Supplementary material for: σE of Streptomyces coelicolor can function both as a direct activator or repressor of transcription
Source: Commun Biol. 2024 Jan 6;7:46. doi: 10.1038/s42003-023-05716-y (PMC10771440; doi:10.1038/s42003-023-05716-y)
Supplement: Supplementary file 10 — Reporting Summary [file 42003_2023_5716_MOESM10_ESM.pdf]

nature portfolio

Corresponding author(s): Jiří Pospíšil and Jiří Vohradský

Last updated by author(s): Dec 5, 2023

Reporting Summary

Nature Portfolio wishes to improve the reproducibility of the work that we publish. This form provides structure for consistency and transparency in reporting. For further information on Nature Portfolio policies, see our Editorial Policies and the Editorial Policy Checklist.

Statistics

For all statistical analyses, confirm that the following items are present in the figure legend, table legend, main text, or Methods section.

n/a	Confirmed	
☐	☒	The exact sample size (*n*) for each experimental group/condition, given as a discrete number and unit of measurement
☐	☒	A statement on whether measurements were taken from distinct samples or whether the same sample was measured repeatedly
☐	☒	The statistical test(s) used AND whether they are one- or two-sided<br>*Only common tests should be described solely by name; describe more complex techniques in the Methods section.*
☐	☒	A description of all covariates tested
☐	☒	A description of any assumptions or corrections, such as tests of normality and adjustment for multiple comparisons
☐	☒	A full description of the statistical parameters including central tendency (e.g. means) or other basic estimates (e.g. regression coefficient) AND variation (e.g. standard deviation) or associated estimates of uncertainty (e.g. confidence intervals)
☐	☒	For null hypothesis testing, the test statistic (e.g. *F*, *t*, *r*) with confidence intervals, effect sizes, degrees of freedom and *P* value noted<br>*Give P values as exact values whenever suitable.*
☒	☐	For Bayesian analysis, information on the choice of priors and Markov chain Monte Carlo settings
☒	☐	For hierarchical and complex designs, identification of the appropriate level for tests and full reporting of outcomes
☒	☐	Estimates of effect sizes (e.g. Cohen's *d*, Pearson's *r*), indicating how they were calculated

Our web collection on statistics for biologists contains articles on many of the points above.

Software and code

Policy information about availability of computer code

Data collection	no software was used
Data analysis	Code for promoter binding motif analysis is available on: https://github.com/cas-bioinf/Scoe_SigE_ChIP-seq

For manuscripts utilizing custom algorithms or software that are central to the research but not yet described in published literature, software must be made available to editors and reviewers. We strongly encourage code deposition in a community repository (e.g. GitHub). See the Nature Portfolio guidelines for submitting code & software for further information.

Data

Policy information about availability of data

All manuscripts must include a data availability statement. This statement should provide the following information, where applicable:

- Accession codes, unique identifiers, or web links for publicly available datasets
- A description of any restrictions on data availability
- For clinical datasets or third party data, please ensure that the statement adheres to our policy

ChIP-seq data and technical details were deposited at ArrayExpress (https://www.ebi.ac.uk/biostudies/arrayexpress) under the accession number E-MTAB-10987. Visualization of ChIP-seq results is available at https://cas-bioinf.github.io/Scoe_SigE_ChIP-seq/. The mass spectrometry proteomics data were deposited to the ProteomeXchange Consortium via the PRIDE [1] partner repository with the dataset identifier PXD041877. Source data that are used in charts are available in Supplementary Data 5.

Research involving human participants, their data, or biological material

Policy information about studies with human participants or human data. See also policy information about sex, gender (identity/presentation), and sexual orientation and race, ethnicity and racism.

Reporting on sex and gender	Not applicable.
Reporting on race, ethnicity, or other socially relevant groupings	Not applicable.
Population characteristics	Not applicable.
Recruitment	Not applicable.
Ethics oversight	Not applicable.

Note that full information on the approval of the study protocol must also be provided in the manuscript.

Field-specific reporting

Please select the one below that is the best fit for your research. If you are not sure, read the appropriate sections before making your selection.

☒ Life sciences ☐ Behavioural & social sciences ☐ Ecological, evolutionary & environmental sciences

For a reference copy of the document with all sections, see nature.com/documents/nr-reporting-summary-flat.pdf

Life sciences study design

All studies must disclose on these points even when the disclosure is negative.

Sample size	All relevant information is in the manuscript and supplemental files.
Data exclusions	Not applicable.
Replication	The experiments were conducted in several biological replicates, specified in each case in the relevant Figure legend.
Randomization	Not applicable.
Blinding	Not applicable.

Reporting for specific materials, systems and methods

We require information from authors about some types of materials, experimental systems and methods used in many studies. Here, indicate whether each material, system or method listed is relevant to your study. If you are not sure if a list item applies to your research, read the appropriate section before selecting a response.

Materials & experimental systems

n/a	Involved in the study	
☐	☒	Antibodies
☒	☐	Eukaryotic cell lines
☒	☐	Palaeontology and archaeology
☒	☐	Animals and other organisms
☒	☐	Clinical data
☒	☐	Dual use research of concern
☒	☐	Plants

Methods

n/a	Involved in the study	
☐	☒	ChIP-seq
☒	☐	Flow cytometry
☒	☐	MRI-based neuroimaging

Antibodies

Antibodies used	Mouse monoclonal antibody against RNAP beta subunit (GeneTex, cat. no: GTX12087, clone 8RB13) and secondary antibody conjugated with HRP (Sigma-Aldrich, cat. no.: A9044-2ML). SigE-HA cellular level was detected by a high affinity monoclonal anti-HA antibody conjugated with HRP (Roche, cat. no.: 12013819001).

Validation

https://www.genetex.com/Product/Detail/RNA-polymerase-beta-antibody-8RB13/GTX12087
https://www.sigmaaldrich.com/CZ/en/product/sigma/a9044
https://www.sigmaaldrich.com/CZ/en/product/roche/12013819001

Plants

Seed stocks	Not applicable.
Novel plant genotypes	Not applicable.
Authentication	Not applicable.

ChIP-seq

Data deposition

☒ Confirm that both raw and final processed data have been deposited in a public database such as GEO.

☒ Confirm that you have deposited or provided access to graph files (e.g. BED files) for the called peaks.

Data access links<br>*May remain private before publication.*	ChIP-seq data and technical details were deposited at ArrayExpress under the accession number E-MTAB-10987 (https://www.ebi.ac.uk/biostudies/arrayexpress/studies/E-MTAB-10987?query=E-MTAB-10987).
Files in database submission	ERR6808227.fastq.gz ERR6808228.fastq.gz ERR6808229.fastq.gz ERR6808230.fastq.gz ERR6808231.fastq.gz ERR6808232.fastq.gz ERR6808233.fastq.gz ERR6808234.fastq.gz ERR6808235.fastq.gz ERR6808236.fastq.gz ERR6808237.fastq.gz
Genome browser session<br>(e.g. UCSC)	Visualization of ChIP-seq results is available at https://cas-bioinf.github.io/Scoe_SigE_ChIP-seq/

Methodology

Replicates	3-4 biological replicates
Sequencing depth	The total number of reads: 550 million.<br>Length of reads: 84
Antibodies	anti-HA high affinity antibody (Roche, cat. no.: 11867423001)

Peak calling parameters

E2-E4macs2-log.txt:

Dataset name: E2-E4macs2-log.txt

Created Wed Jul 17 15:49:40 CEST 2019

Created with Chipster 3.12.3

Created with operation: ChIP- and DNase-seq / Find peaks using MACS2

Parameter Input file format: BAM

Parameter Mappable genome size: User specified

Parameter User specified mappable genome size: 9e6

Parameter q-value cutoff: 0.01

Parameter Read length: 0

Parameter Keep duplicate reads: auto

Parameter Build peak model: yes

Parameter Bandwidth: 300

Parameter Extension size: 200

Parameter Upper M-fold cutoff: 30

Parameter Lower M-fold cutoff: 10

Parameter Call broad peaks: no

Dataset name: C1-C4macs2-log.txt

Created Thu Jul 18 08:57:33 CEST 2019

Created with Chipster 3.12.3

Created with operation: ChIP- and DNase-seq / Find peaks using MACS2

Parameter Input file format: BAM

Parameter Mappable genome size: User specified

Parameter User specified mappable genome size: 1e9

Parameter q-value cutoff: 0.01

Parameter Read length: 0

Parameter Keep duplicate reads: auto

Parameter Build peak model: yes

Parameter Bandwidth: 300

Parameter Extension size: 200

Parameter Upper M-fold cutoff: 30

Parameter Lower M-fold cutoff: 10

Parameter Call broad peaks: no

Data quality	FastQC (https://www.bioinformatics.babraham.ac.uk/projects/fastqc/)
Software	Chipster (https://chipster.metacentrum.cz)
